# Protocol to characterize tumor microenvironment in a diffuse midline glioma mouse model using multi-omics approach on sequential sections

**DOI:** 10.1016/j.xpro.2026.104801

**Published:** 2026-08-24

**Authors:** Raphaël Collot, Celina Honhoff, Cristian Ruiz-Moreno, Ravian L. van Ineveld, Anne C. Rios

**Affiliations:** 1 Princess Máxima Center for pediatric oncology, Utrecht, the Netherlands; 2 Oncode Institute, Utrecht, the Netherlands; 3 Department of Biology, Faculty of Science, Utrecht University, Utrecht, the Netherlands

**Keywords:** Antibody, Cancer, Microscopy, RNAseq, Single Cell

## Abstract

Diffuse midline glioma (DMG) tumors form complex tumor-immune microenvironments with marked cellular heterogeneity. Here, we present a protocol to generate orthotopic DMG tumors in immunocompetent neonatal mice and to spatially characterize the tumor ecosystem using a multi-omics workflow on sequential brain sections. We detail procedures for brain freezing, cryosectioning, single-nuclei RNA sequencing, and Visium spatial transcriptomics. We then describe steps for cyclic immunofluorescence and integrative computational analysis to resolve cellular states and spatial organization.

For complete details on the use and execution of this protocol, please refer to Collot et al.^1^

## Highlights

• Instructions for generating a neonatal syngeneic DMG mouse model in immunocompetent mice
• Steps for processing sequential brain sections for snRNA-seq, Visium, and cyclic IF
• Guidance on cyclic-IF imaging and open-source image co-registration

## Abstract (editor highlights)

Publisher’s note: Undertaking any experimental protocol requires adherence to local institutional guidelines for laboratory safety and ethics.

## Graphical abstract

**Figure:**
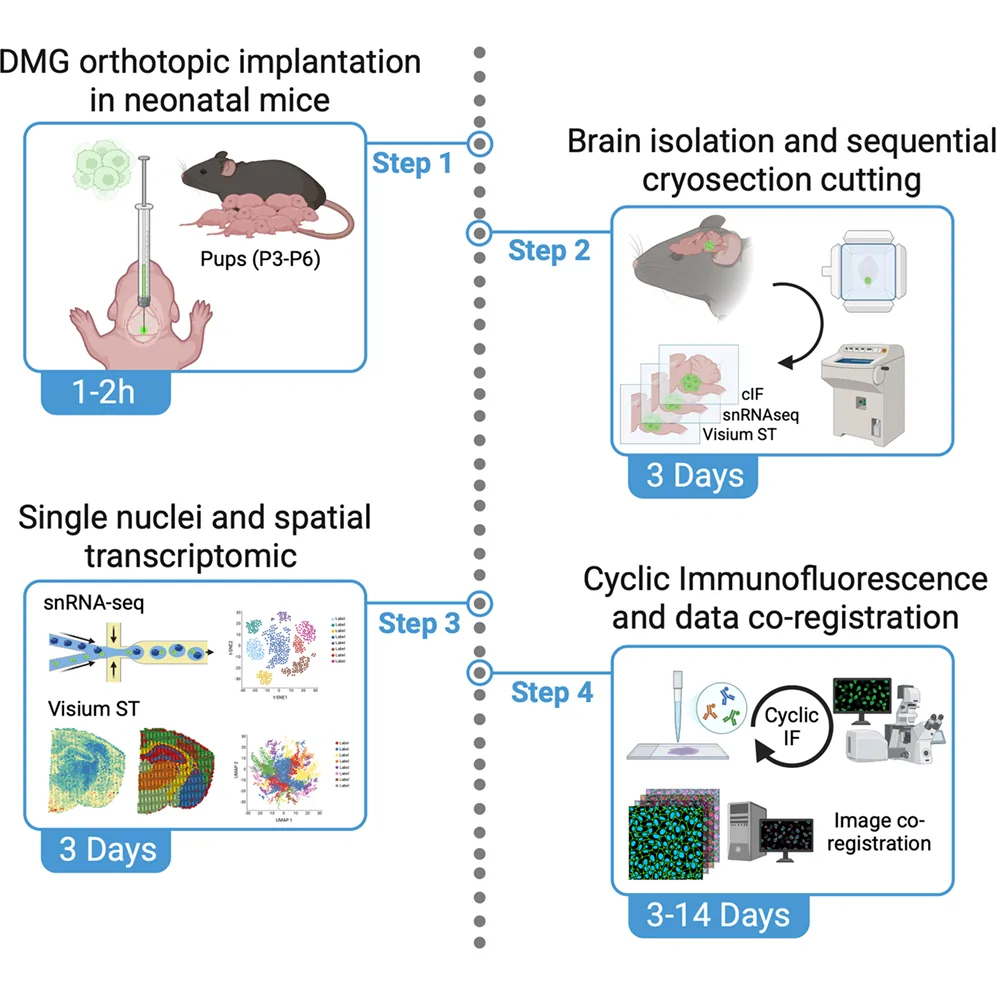

## Before you begin

Spatial multi-omics profiling enables mapping of transcriptional and protein-level heterogeneity within the tumor microenvironment and allows integration of cellular states with spatial organization. Integration of single-nuclei RNA sequencing (snRNA-seq) with spatial transcriptomics further enables cell-type-resolved interpretation of regional gene expression patterns within intact tumor tissue.

In this protocol, we describe the specific steps to generate a syngeneic pontine diffuse midline glioma (DMG) model in immunocompetent neonatal mice and to process fresh-frozen tumor bearing brains for sequential multi-omics analyses. Note that this model has previously been described in adult mice by Chatinier *et al*.^2^ We perform single-nuclei RNA sequencing using the 10× Genomics Chromium 3′ platform, Visium Spatial Gene Expression on adjacent cryosections, and cyclic immunofluorescence for protein-level validation. This workflow allows integrative analysis of matched sections from the same tumor to resolve spatial cellular heterogeneity, as described in Collot *et al*.^1^ The workflow has been successfully applied to multiple independent biological replicates, yielding reproducible tissue processing, imaging quality, and multi-omics data generation. Although we focus on a pontine DMG model, the tissue processing workflow can be adapted to other orthotopic brain tumor models with optimization.

Before beginning with the stereotactic injection of DMG cells in neonatal mice, contact the lead authors from Ho *et al.*^3^ to receive the 3D printing file for their anesthesia compatible injection mold. If the 3D-printed mold is not available, neonatal mice may be stabilized using alternative setups, such as a carved-out Styrofoam holder,^4^ to ensure accurate and reproducible head positioning.

Before beginning Visium Spatial Gene Expression, review the 10× Genomics Visium Spatial Gene Expression User Guide for fresh-frozen tissue, including instructions for tissue embedding, cryosectioning onto a Visium Gene Expression Slide, H&E staining or immunofluorescence imaging, permeabilization optimization, cDNA library construction, and sequencing. Tissue-specific permeabilization time optimization is required, as mRNA released during permeabilization binds to spatially barcoded capture probes on the slide. Downstream analysis requires the use of Space Ranger and Loupe Browser.

Before beginning single-nuclei RNA sequencing using the 10x Genomics Single cell 3′ platform, review the Chromium Single Cell 3′ Reagent Kits User Guide, including guidance on nuclei quality assessment, GEM generation, reverse transcription, library construction, and sequencing requirements. Ensure that nuclei isolation conditions are optimized for frozen brain tissue to preserve RNA integrity and minimize debris and doublets.

Finally for the cyclic immunofluorescence, ensure to have the appropriate microscope setup, data storage capacity and workstations to run the co-registration pipeline in Python and basic imaging analyses software like QuPath.

### Innovation

This protocol advances tumor microenvironment characterization by integrating transcriptomic and proteomic readouts on sequential sections, optimizing workflows for mouse brain tissue. We modified the injection age of the DMG mouse model to an early post-natal window to better mimic the tumor developmental time frame of DMG^5^^,^^6^ and optimized the nuclei isolation procedure to yield high quality nuclei from fresh frozen mouse brain cryosections. Finally, we optimized the cyclic immunofluorescence protocol to avoid the need for specialized equipment during imaging and developed a custom co-registration pipeline to align the imaging rounds without the need for commercial software.

### Institutional permissions

All murine experiments were conducted in compliance with the Animal Welfare Committee of the Princess Maxima Center and both local and international regulations. Mice were housed at 45%–65% humidity, 20.5–23.5^◦^C and 12-h light/12-h dark cycle, in Specific Opportunistic Pathogen Free (SOPF) conditions using individually ventilated cages and sterile food and water ad libitum. Time-pregnant C57BL/6N mice were used for timed breeding and neonatal injection within this study. Researchers intending to apply this protocol must obtain approval from their respective institutional animal care committees and ensure compliance with all applicable regulatory standards prior to conducting any animal experiments.

### Preparation of timed breeding pairs

**Timing: ∼10 min (setup; additional time required for gestation)**

This step describes the setup of timed breeding pairs to obtain neonatal pups for stereotactic injections.

1. Set up timed breeding pairs approximately 3 weeks prior to the intended injection date to ensure availability of neonatal pups at the required postnatal stage. House one male with one or two females.
2. Separate females into individual cages two days later.
3. Check for pregnancy after two weeks by palpation of the embryos or visual check of the belly.
4. Schedule the stereotaxic injection between post-natal day 3 to 5.

***Note:*** Set up enough breeding pairs to obtain the desired number of pups for the experiment. Typically, a maximum of six pups per litter can be injected. For C57BL/6N mice, litters contain approximately 5–8 pups, although this may vary between colonies. We recommend consulting strain-specific breeding information and your institutional animal facility to estimate expected litter sizes and plan breeding cohorts accordingly. In addition, as maternal performance and pup survival can be more variable in first-time dams, we recommend using dams that have successfully reared a previous litter.

### Preparation of IUE-derived murine DMG cell line

**Timing: ∼20–30 min**

This step describes the preparation of a single-cell suspension from IUE-derived DMG spheroids for stereotactic injection into neonatal mice.

5. Collect IUE-derived DMG spheroids in a 15 mL conical tube and centrifuge for 5 min at 300 × *g* at room temperature.
6. Carefully remove the supernatant without disturbing the pellet.
7. Add approximately 10 volumes of Accutase dissociation reagent relative to the pellet volume and gently resuspend until a homogeneous suspension is obtained.
8. Incubate for 5 min at 37°C.
9. Add 4.5 mL of DMG working medium to inactivate Accutase and gently resuspend the cells.
10. Determine cell concentration and viability using a Countess Cell Counter or any other appropriate counting method.
11. Centrifuge the cells for 5 min at 300 × *g* at 4°C.
12. Remove the supernatant and resuspend the cell pellet in cold PBS to obtain a final concentration of 150,000 cells/μL, allowing injection of 300,000 cells in 2 μl PBS per pup.

***Note:*** Prepare approximately 30% excess volume to account for handling losses and dead volume in the Hamilton syringe.

**CRITICAL:** Proceed with *in-vivo* injections only if cell viability exceeds 80% and sufficient cells are available for all planned injections. Although higher cell viability may reduce the risk of syringe clogging, we have not observed major differences in tumor growth when injecting cells with viabilities ranging from 80% to 100%. Keep the concentrated cell suspension on ice and perform injections within 1 h to preserve cell viability.

### Preparation of buffers and instruments for stereotactic injection

**Timing: ∼30 min**

**Figure 1:**
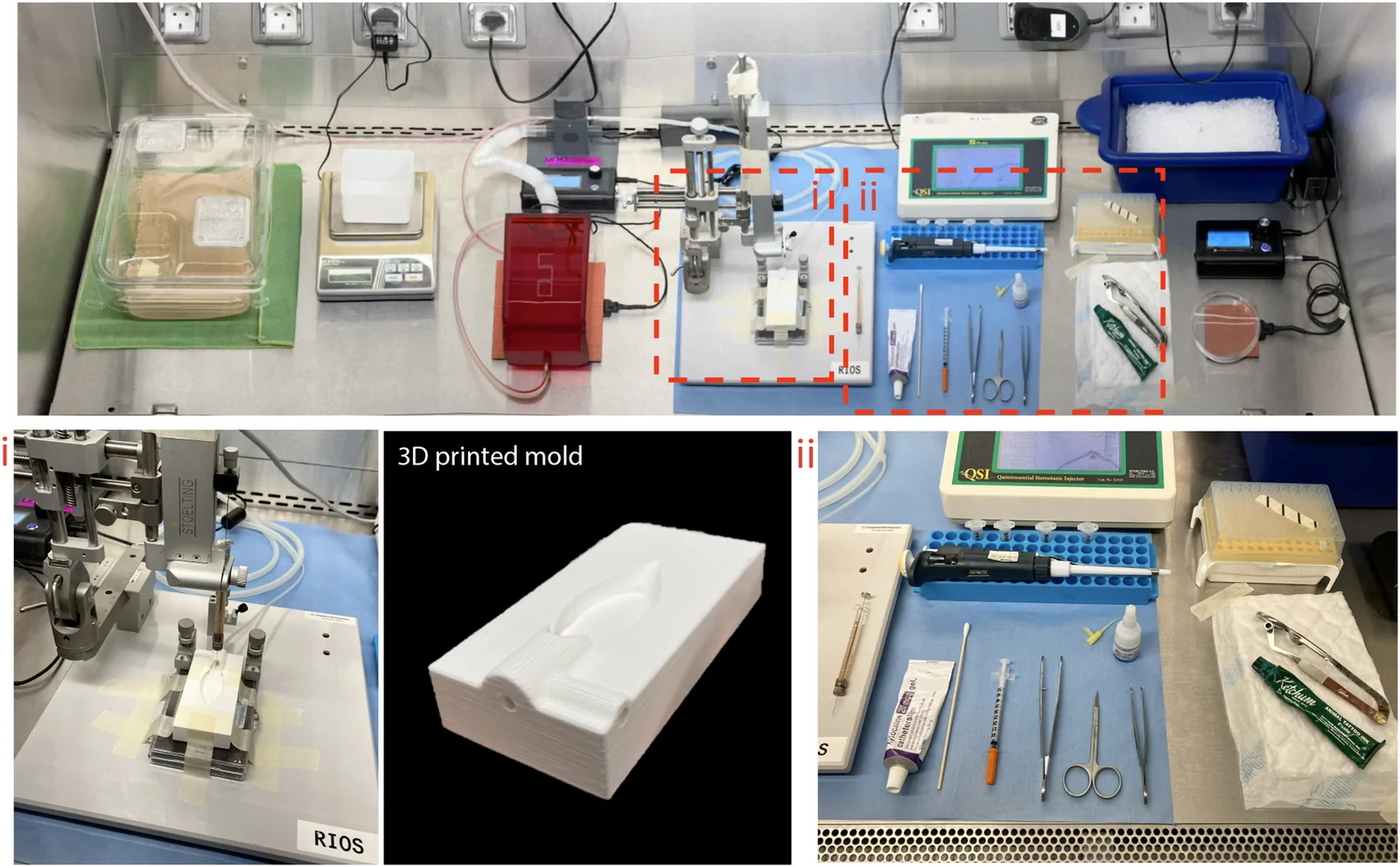
Stereotactic injection station for neonatal mice Prepare all the necessary tools and equipment for the tumor cell injection (top image). Detailed image (i) of the 3D printed pup injection mold placed under the stereotactic frame. Detailed image (ii) of the instruments necessary for the surgical procedure.
14. Sterilize surgical instruments using a bead sterilizer or by immersion in 70% ethanol. Allow instruments to cool and dry before use.
15. Disinfect all working surfaces, stereotactic equipment, and consumables using 70% ethanol prior to starting the procedure.

***Note:*** Maintain a sterile working environment throughout the procedure to minimize the risk of infection.

13. Prepare all required buffers and surgical materials as described in the materials and equipment section (Figure 1).

### Preparation of buffers and instruments for intact brain isolation and snap freezing

**Timing: ∼1 h**

16. Prepare all required buffers and surgical materials as described in the materials and equipment section.
17. Pre-chill phosphate-buffered saline (PBS) on ice for intracardiac perfusion and for temporary storage of the extracted brain.
18. Set up the snap-freezing station by gathering cryomolds, labels, OCT embedding medium, forceps, containers, isopentane, and dry ice (Figure 2).

**Figure 2:**
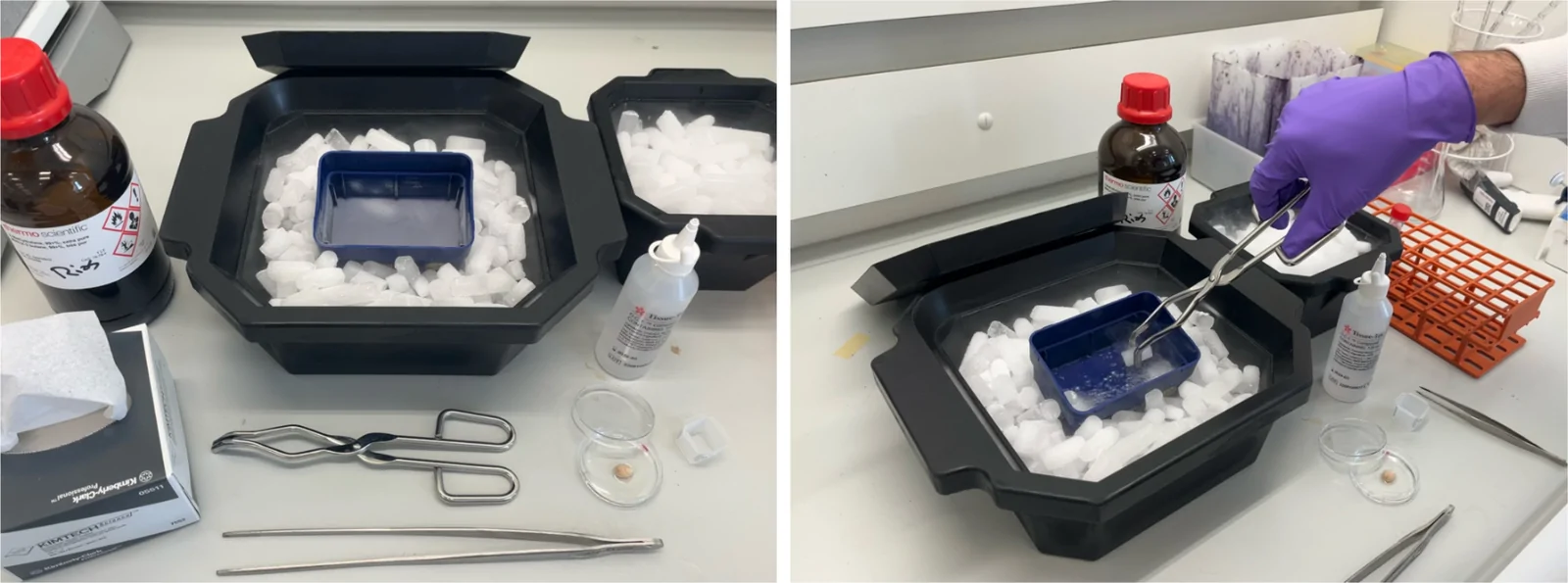
Mouse brain snapfreezing station Prepare all the necessary tools and containers (left) before adding isopentane to the pre-cooled container on dry ice and adding dry ice blocks to the isopentane for further cooling (right)
19. Pre-chill OCT embedding medium at 4°C for at least 1 h prior to use. Cool isopentane on dry ice for approximately 5 min before snap freezing until it reaches a semi-frozen, slushy consistency.
20. Prepare a container filled with dry ice to store embedded samples immediately after freezing before transferring them to a −80°C freezer.

**CRITICAL:** Ensure that all materials are prepared in advance and that the snap-freezing workflow is performed rapidly after brain extraction to preserve RNA integrity and tissue morphology.

***Note:*** Proper cooling of isopentane is essential for optimal tissue preservation and to prevent ice crystal formation. Liquid nitrogen can be used as an alternative to dry ice to cool down the isopentane.

### Preparation of buffers and utensils for single nuclei isolation

**Timing: 45–60 min**

This step describes the preparation of chilled buffers, tubes, and equipment required for single-nucleus isolation from fresh-frozen mouse brain cryosections prior to fluorescence-activated nuclei sorting and Chromium Single Cell 3′ library preparation. All steps should be performed on ice or at 4°C to preserve nuclei integrity and RNA quality.

21. Pre-chill a fixed-angle centrifuge to 4°C and place all required reagents and consumables on ice (Figure 3). Chill DNA LoBind tubes and one Dounce grinder set per sample before starting the procedure.

**Figure 3:**
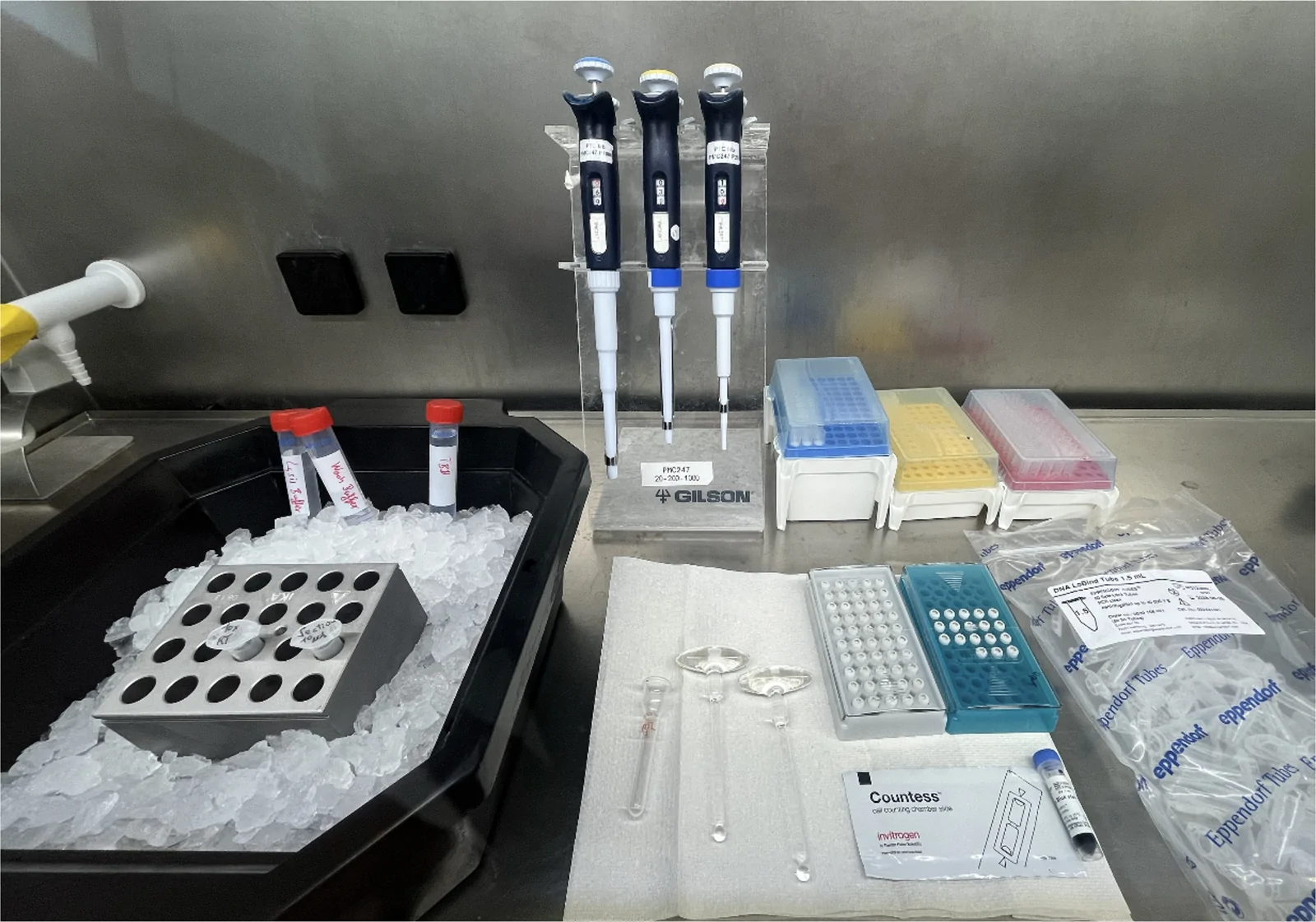
Single-nuclei isolation setup Prepare all the necessary tools and equipment in a clean flow hood. Keep the buffers and reagents on ice and do not introduce any RNases. One set of dounce grinder is needed per sample.
22. Prepare fresh Tris-based buffer as described in the materials and equipment section and keep it on ice.
23. Prepare nuclei lysis buffer and wash buffer immediately before use as described in the materials and equipment section and keep the solution on ice.
24. Prepare chilled 10× RT buffer for Chromium Single Cell Gene Expression 3′ reagents for each sample, but do not add RT Enzyme C at this stage.
  a. Vortex and briefly centrifuge the RT reagent, template switch oligo, and Reducing Agent B before use.
  b. Adjust the final pre-sort volume to 71.7 μL with nuclease-free water if needed.
  c. After sorting, add RT Enzyme C to bring the final volume to 80 μL immediately before chip loading.
25. Prepare 70 μm and 40 μm strainers, extended-length pipette tips, and ice-cold work surfaces for nuclei handling and filtration.

**CRITICAL:** Keep all buffers, reagents, tubes, and tools cold throughout the entire procedure. Minimize the time between nuclei isolation, sorting, and Chromium chip loading to preserve RNA integrity and reduce debris and doublets.

## Key resources table

**Table:** 

REAGENT or RESOURCE	SOURCE	IDENTIFIER
**Antibodies**		

Rabbit anti-H3K27M IgG antibody (1:400)	Thermo Fisher Scientific	Cat# MA5-27916; RRID: AB_2744969
Rabbit anti-PDGFRA IgG antibody (1:400)	Cell Signaling	Cat#3174
Goat anti-IBA1 IgG antibody (1:300)	Novus	Cat# NB100-1028; RRID: AB_521594
Rabbit anti-IGSF11 IgG antibody (1:100)	Thermo Fisher Scientific	Cat# 703178
Rabbit anti-VISTA IgG antibody (1:200)	Cell Signaling	Cat# 54979, RRID: AB_2799474
Rat anti-TREM2 IgG2b antibody (1:100)	Biotechne	Cat# MAP17291
Mouse anti-CD206 IgG2b antibody (1:100)	Biotechne	Cat# MAP25341
Mouse anti-CD44 IgG2b antibody (1:100)	BD Biosciences	Cat#555476
Rat anti-CD31 IgG2a antibody (1:200)	BD Biosciences	Cat# 553370; RRID: AB_396660

**Chemicals, peptides, and recombinant proteins**		

DAPI (1:2000)	Thermo Fisher Scientific	Cat# D3571; RRID: AB_2307445
Low Melting Point (LMP) agarose	Invitrogen	Cat# 16520-050
Donkey serum	Merck	Cat# S30-100ml
Slowfade Gold Antifade	Invitrogen	Cat# S36936
Tissue-Tek O.C.T Compound	Sakura	Cat# 4583
Accutase Cell detachment medium	Thermo Fisher Scientific	Cat#00-4555-56
Isopentane	Merck	Cat#277258
Isoflurane	Veterinary Technics	Cat#171003
Buprenorphine	Covetrus	Cat#1224097
Lidocaine Solution	Covetrus	Cat#4009129
Lidocaine Cream	BENU	Cat#1064237
Ketamine	Covetrus	Cat#1201684
Medetomidine	Covetrus	Cat#1203274
Glycine	Sigma-Aldrich	Cat#219517
Triton X100	Sigma-Aldrich	Cat#282103
Ammonium Chloride	Sigma-Aldrich	Cat#254134
Maleimide	Sigma-Aldrich	Cat#915343
Guanidinum Chloride	Sigma-Aldrich	Cat#G3272
Urea	Sigma-Aldrich	Cat#U1250
TCEP	Sigma-Aldrich	Cat#C4706
MACSima Running buffer	Miltenyi Biotec	Cat#130-121-565
Lunaphore Elution Buffer Kit	Lunaphore	Cat#BU07
RNasin Ribonuclease Inhibitor	Promega	Cat#N2111
BSA	Sigma-Aldrich	Cat#A9418
Neurobasal™-A Medium	Thermo Fisher	Cat#10888-022
Advanced DMEM/F-12 (1×)	Thermo Fisher	Cat#12634-010
HEPES Buffer Solution (1 M)	Thermo Fisher	Cat#15630-056
MEM Non-Essential Amino Acids Solution (100×)	Thermo Fisher	Cat#11140-035
GlutaMAX™ Supplement (100×)	Thermo Fisher	Cat#35050-038
Sodium Pyruvate (100 mM)	Thermo Fisher	Cat#11360-070
Primocin	InvivoGen	Cat#ant-pm-05
B-27™ Supplement minus vitamin A (50×)	Thermo Fisher	Cat#12587-010
Recombinant Human FGF-basic	PeproTech	Cat#100-18B
Recombinant Human EGF	PeproTech	Cat#AF-100-15
Recombinant Human PDGF-AA	PeproTech	Cat#AF-100-13A
Recombinant Human PDGF-BB	PeproTech	Cat#AF-100-14B
Heparin Solution	STEMCELL Technologies	Cat#07980
Paraformaldehyde	Sigma-Aldrich	Cat#441244

**Critical commercial assays**		

Single Cell Gene Expression 3’v3.1	10×Genomics	Cat#PN-1000269
Visium Spatial Gene Expression Slide & Reagent Kit	10×Genomics	Cat#PN-1000187
Rneasy mini kit	QIAGEN	Cat#74104
Bioanalyzer High Sensitivity RNA Analysis Kit	Agilent	Cat#5067-1513

**Experimental models: Cell lines**		

Murine IUE derived DMG 24B7 spheroids. C57BL/6NCrl background	Gift from Esther Hulleman’s group, Princess Máxima Center for Pediatric Oncology, Utrecht, the Netherlands. Generated by Timothy N Phoenix’s lab, Cincinnati Children’s Hospital Medical Center, Ohio, USA.	N/A

**Experimental models: Organisms/strains**		

Mouse: C57BL/6NCrl (C57BL/6), neonatal female and male	Charles River	Strain# 475

**Software and algorithms**		

Seurat version 4.0	https://satijalab.org/seurat/	RRID: SCR_016341
DoubletFinder version 2.0.3	https://github.com/chris-mcginnis-ucsf/DoubletFinder	RRID: SCR_018771
Numbat version 1.2.3	https://github.com/kharchenkolab/numbat	RRID: SCR_019207
SeuratWrappers version 0.3.0	https://github.com/satijalab/seurat-wrappers	RRID: SCR_022555
SCpubR version 1.1.2	https://github.com/enblacar/SCpubr	RRID: SCR_021139
DittoSeq version 1.11.0	https://github.com/dtm2451/dittoSeq	N/A
Scanpy version 1.9.3	https://scanpy.readthedocs.io/en/stable/	RRID: SCR_018139
Cell2location version 0.1.3.	https://cell2location.readthedocs.io/en/latest/	RRID: SCR_024859
Python version 3.9	https://www.python.org	RRID: SCR_008394
R version 4.0.2	https://www.r-project.org	RRID: SCR_001905
QuPath	https://qupath.readthedocs.io/en/0.6/	RRID: SCR_018257

**Other**		

Countess Automated Cell Counter	Thermo Fisher	Cat#AMQAX2000
Countess Automated Cell Counting Chamber Slides	Thermo Fisher	Cat#C10228
Stereotaxic Frame	Stoelting	Cat#53311
Hamilton micro syringe	Hamilton	Cat#87930
Vetbond Surgical Glue	WPI	Cat#7341
Kimtech Wipes	Kimberly-Clark	Cat#7552
Peel-A-Wat Disposable Histology Mold	Ted Pella Inc.	Cat#27110
CryoStar NX50 Cryostat	Thermo Fisher	Cat#957240
Agilent 2100 Bioanalyzer	Agilent	NA
Superfrost Plus Adhesion Microscope Slides	Epredia	Cat#J1800AMNZ
Hydrophobic barrier (PAP) Pen	Thermo Fisher	Cat#R3777
Coverslip	Epredia	Cat#BB024060M1
Mounting medium SlowFade Gold	Thermo Fisher	Cat#S36936
Leica Thunder Imager	Leica Microsystems	NA
DNA LoBind tubes	Eppendorf	Cat#003018051
Kimble Dounce tissue grinder set	Merck	Cat#D8938
Flowmi cell strainer 70 μm	Merck	Cat#BAH136800070
Flowmi cell strainer 40 μm	Merck	Cat#BAH136800040
CoolRack	Merck	Cat#CLS432041-EA

## Materials and equipment

**Table:** 4% PFA

Reagent	Final concentration	Amount
Paraformaldehyde (prilled, 95%)	4%	20 g
PBS	–	500 mL
Total	–	500 mL

Store aliquots at −20°C for 6 months, thaw before use and do not freeze-thaw.

**Table:** DMG Base Medium

Reagent	Final concentration	Amount
Neurobasal-A medium	50%	239.5 mL
Advanced DMEM/F12 (1×)	50%	239.5 mL
MEM Non-Essential Amino Acids (100×)	1×	5 mL
GlutaMAX™ Supplement (100×)	1×	5 mL
Sodium Pyruvate (100 mM)	1mM	5 mL
HEPES Buffer Solution (1 M)	0.01 M	5 mL
Primocin	0.1 mg/mL	1 mL
**Total**	–	500 mL

Store at 4°C for up to 3 months.

**Table:** DMG Working Medium

Reagent	Final concentration	Amount
DMG Base Medium	–	49 mL
B-27™ Supplement minus vitamin A (50×)	1×	1 mL
Recombinant Human FGF-basic	20 ng/mL	10 μL
Recombinant Human EGF	20 ng/mL	10 μL
Recombinant Human PDGF-AA	10 ng/mL	5 μL
Recombinant Human PDGF-BB	10 ng/mL	5 μL
Heparin	2 μg/mL	50 μL
**Total**	–	50 mL

Store at 4°C for up to 1 week.

**Table:** cIF Quenching Buffer for 4 glass slides

Reagent	Final concentration	Amount
Glycine	0.1 M	7.5 mg
Nuclease-free water	–	1 mL
**Total**	–	1 mL

Prepare the solution freshly before use.

**Table:** cIF Permeabilization Buffer for 4 glass slides

Reagent	Final concentration	Amount
Triton X-100	0.2%	20 μL
PBS	–	9.98 mL
**Total**	–	10 mL

Store at room temperature for 2 weeks.

**Table:** cIF Blocking Buffer for 4 glass slides

Reagent	Final concentration	Amount
NH_4_Cl	100 mM	26.74 mg
Maleimide	150 mM	72.80 mg
Donkey serum	10%	0.5 mL
PBS	–	4.5 mL
**Total**	–	5 mL

Store at 4°C and prepare freshly every 2–3 days.

***Alternatives:*** Use MACSima Running Buffer supplemented with 10% donkey serum.

**Table:** cIF Staining Buffer for 4 glass slides

Reagent	Final concentration	Amount
NH_4_Cl	100 mM	53.49 mg
Donkey serum	5%	0.5 mL
PBS	–	9.5 mL
**Total**	–	10 mL

Store at 4°C and prepare freshly every 2–3 days.

***Alternatives:*** Use MACSima Running Buffer supplemented with 5% donkey serum.

**Table:** cIF Elution Buffer for 4 glass slides

Reagent	Final concentration	Amount
Glycine	0.5 M	37.56 mg
Guanidinium chloride	3 M	287.2 mg
Urea	3 M	180 mg
TCEP	40 mM	11.48 mg
Nuclease-free water	–	1 mL
**Total**	–	1 mL

Prepare freshly before use.

***Alternatives:*** Use Lunaphore Elution Buffer Kit. Combine Solution 1 and 2 according to manufacturer protocol.

**Table:** Tris-based buffer (10 mL; sufficient for 2 samples)

Reagent	Stock concentration	Final concentration	Amount
Tris-HCl, pH 7.4	1 M	10.0 mM	100 μL
NaCl	5 M	146.0 mM	292 μL
MgCl2	1 M	21.0 mM	210 μL
CaCl2	1 M	1.0 mM	10 μL
Nuclease-free water	N/A	N/A	9388 μL
**Total**	–	–	10 mL

Prepare the solution freshly before use.

**Table:** Nuclei lysis buffer (4.05 mL; sufficient for 2 samples)

Reagent	Stock concentration	Final concentration	Amount
Tris-based buffer	–	–	3.995 mL
RNasin	40 U/μL	100 U/mL (0.1 U/μL)	10 μL
NP-40	10%	0.1%	40 μL
**Total**	–	–	4.05 mL

Prepare the solution freshly before use and keep at 4°C.

**Table:** Wash buffer (5.025 mL; sufficient for 2 samples)

Reagent	Stock concentration	Final concentration	Amount
Tris-based buffer	–	–	3,995 mL
PBS + BSA	10% BSA stock	2% BSA	1 mL
RNasin	40 U/μL	200 U/mL (0.2 U/μL)	25 μL
**Total**	–	–	5.020 mL

Prepare the solution freshly before use and keep at 4°C.

**Table:** 10× RT buffer for sorting (per sample)

Reagent	Stock concentration	Final concentration	Amount
RT reagent	per 10× reagent kit	manufacturer mix	20 μL
TSO	per 10× reagent kit	manufacturer mix	3.1 μL
Reducing Agent B	per 10× reagent kit	manufacturer mix	2 μL
Nuclease-free water	–	adjust as needed	variable
**Pre-sort total**	–	–	71.7 μL
RT Enzyme C	per 10× reagent kit	added after sorting	8.3 μL
**Final total after sorting**	–	–	80 μL

Prepare the solution freshly before use and keep at 4°C.

**Table:** DAPI staining for nuclei sorting

Reagent	Final concentration	Amount
DAPI	3 μM	2.5 μL per 500 μL nuclei suspension

Prepare the solution freshly before use.

## Step-by-step method details

### Neonatal stereotaxic injection of DMG tumor cells

**Timing: ∼30 min per litter**

These steps describe the procedure for anesthesia, stereotaxic injection, wound closure, and post-operative recovery of P3-P5 mouse pups for intracranial DMG cell transplantation.

1. Selection, assessment, and preparation of neonatal pups for stereotaxic injection.
  a. Count the total number of pups in the litter and assess their overall health and size relative to standard growth curves.
  b. Select pups for injection based on strain-specific guidelines and maternal behavior: use up to 6 pups for C57BL/6 strain.
    ***Note:*** To minimize bias, select equal numbers of male and female pups for injection, where possible.
  c. Euthanize pups that will not be injected by inhalation anesthesia overdose, followed by decapitation.
    **CRITICAL:** If non-injected pups remain in cage or if too many injected pups are returned to cage, dam is more likely to reject injected pups.
  d. Weigh each selected pup individually using a precision balance and record the weight for buprenorphine dosing (0.05 mg/kg).

2. Induction of general anesthesia and administration of systemic analgesia.
  a. Place the pup in an induction chamber positioned on a pre-warmed heating pad to maintain body temperature.
  b. Deliver 4% isoflurane in oxygen for 4 min.
  c. Confirm anesthesia by absence of response to gentle toe pinch.
  d. Administer buprenorphine (0.05 mg/kg) subcutaneously using the pup’s recorded weight.

**Figure 4:**
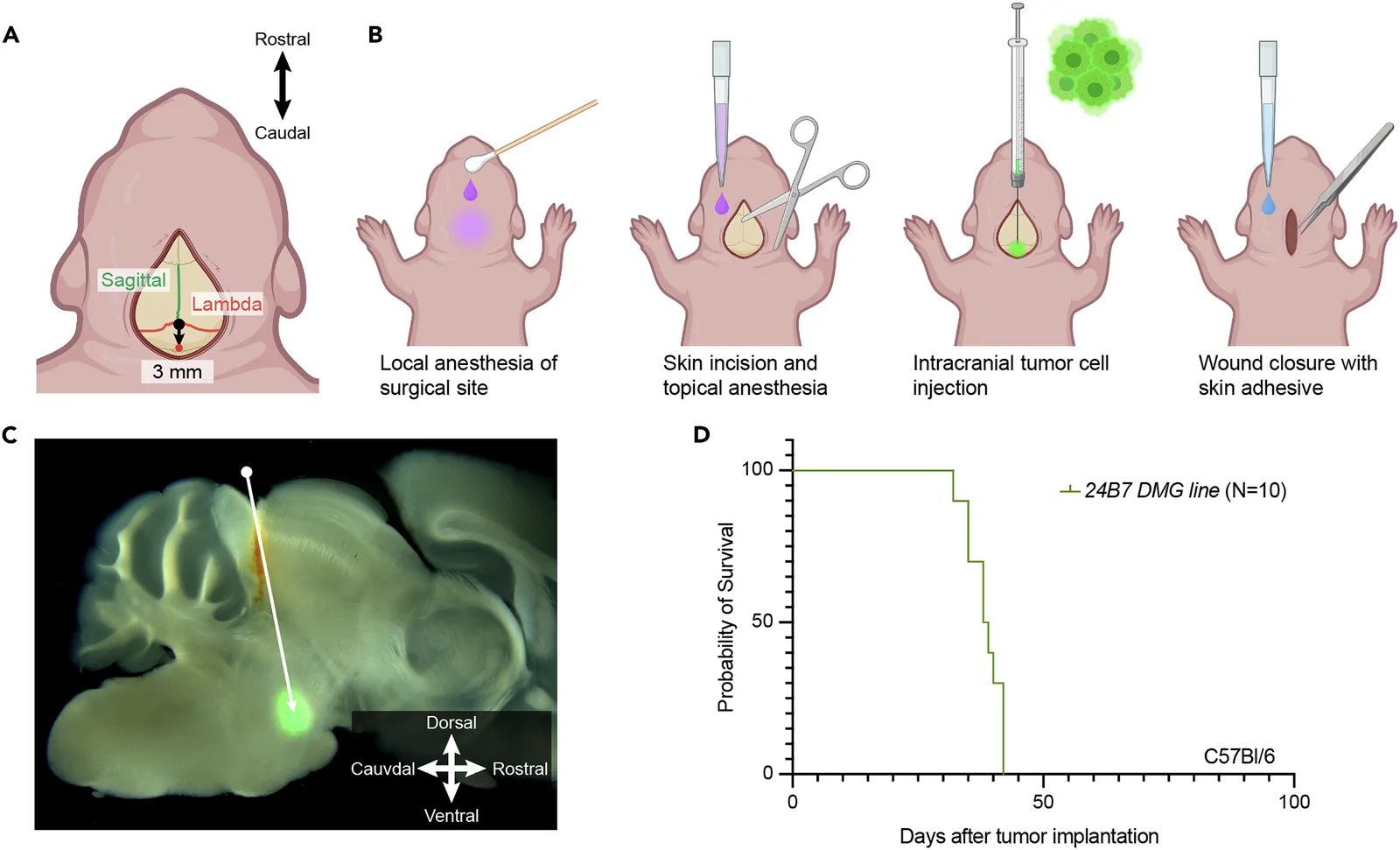
Generation of DMG syngeneic mouse model (A) Injection location (red dot) 3 mm posterior to lambda and 0 mm lateral to the midline. (B) Sequential steps during the surgical procedure. (C) Representative image of DMG tumor growth (green signal) in the pontine area following a 3.5 mm deep injection at neonatal stage. (D) Kaplan-Meier survival curves of immunocompetent C57BL/6 mice allografted with 24B7 DMG line.
3. Positioning of the pup in the stereotaxic frame and local surgical preparation (Figure 1).
  a. Place the 3D-printed neonatal stabilization mold into the stereotaxic frame and connect it to the isoflurane delivery system to maintain continuous anesthesia.
  b. Transfer the anesthetized pup from the induction chamber into the mold, ensuring correct alignment of the head and body.
    ***Note:*** The mold is not heated, so the pup is at risk of hypothermia during surgery. Minimize surgery time, keep procedures efficient, and cover the pup (e.g., with your hand) when possible, to retain warmth.
  c. Apply lidocaine cream (20 mg/g) to the scalp and wait 1–2 min for topical anesthetic effect.
  d. Make a small midline incision in the scalp to expose the skull and identify the lambda and sagittal suture (Figure 4).
  e. Apply 50 μl lidocaine solution (10 mg/mL) directly onto the skull to provide additional local anesthesia.
4. Stereotaxic intracranial injection of DMG tumor cells into the posterior pons.
  a. Reduce isoflurane concentration to 3% to maintain stable anesthesia during injection.
  b. Load solution containing 300,000 DMG cells in 2 μl of PBS into a 10 μl Hamilton micro syringe with a 2-pt style needle, carefully removing all air bubbles.
    ***Note:*** Based on Hamilton syringe dead volume and liquid handling, a maximum of 4 pups can be injected without reloading. Ensure the syringe is not clogged before proceeding with the injections.
  c. Position the needle at lambda, then move to the posterior pons coordinates: 3 mm posterior to lambda and 0 mm lateral (midline) (Figure 4).
    ***Note:*** These injection coordinates are based on previously published protocols and have been reproducibly applied by multiple operators without modification.^7^
  d. Lower the needle until it contacts the skull surface.
  e. Pierce the skull using the needle tip by applying gentle downward pressure until the skull loses resistance.
    ***Note:*** Due to the curved surface of the neonatal skull and minor variability in skull shape between pups, ensure the head is consistently stabilized within the mold and the needle is properly positioned perpendicular to the skull to prevent slipping or unintended penetration at an incorrect location.
  f. After penetrating the skull, retract the needle slightly and reset the depth measurement to ensure accurate targeting from the skull surface.
  g. Lower the needle to the target depth (3.5 mm) and inject 2 μl cell suspension at 2 μl/min rate.
  h. Reduce concentration to 1% and wait 30 seconds after injection completion, then slowly retract the needle. Resuspend the cells between each injection by gently tapping the syringe.
    **CRITICAL:** Rapid injection or withdrawal may result in reflux or poor tumor engraftment. Reflux may be visually observed at the injection site and can be minimized by allowing sufficient dwell time before needle retraction.
    ***Note:*** If the injected cells contain a GFP construct, correct injection targeting can be validated by GFP signal (Figure 4C). Alternative readouts like histology or antibody stainings can also be used to confirm successful implantation.

5. Surgical closure of the incision and post-injection identification of pups.
  a. Close the skin incision using surgical glue, ensuring complete sealing of the skin.
  b. If necessary, mark the pup using a micro-tattooing system to enable identification of experimental groups.
6. Post-operative recovery monitoring.
  a. Transfer pup to a Petri dish placed on a pre-warmed heating pad containing a small amount of home cage bedding to retain familiar scent cues during recovery.
  b. Monitor breathing and spontaneous movement until full recovery.
  c. If breathing is delayed, provide gentle tactile stimulation until respiration resumes.
  d. Keep all injected pups together until all littermates have been injected.
7. Return of pups to the dam and observation for maternal care and pup survival.
  a. Return pups to the dam only after they are fully active and breathing regularly.
  b. Observe maternal behavior 2 h post-return, watching for signs of pup rejection: Agitation, excessive carrying of pups, failure to nurse.
  c. If maternal rejection occurs, transfer pups to a foster dam, or euthanize pups using inhalation anesthesia overdose followed by decapitation.

***Note:*** The overall experimental success rate is approximately 80%. Experimental losses are primarily due to post-procedural pup survival and maternal care, whereas successful tumor engraftment is observed in the vast majority of surviving animals.

### Post-operative monitoring and tumor progression assessment

**Timing: 4–6 weeks from day of injection until experimental endpoint**

These steps describe post-operative monitoring, routine health assessment, and tumor progression tracking following neonatal intracranial injection of DMG cells.

8. Post-operative survival and early monitoring of pups.
  a. Perform daily visual monitoring during the first week post-injection to assess survival and general condition.

***Note:*** Minimize disturbance of the cage and litter during monitoring, as increased handling and stress can elevate the risk of maternal rejection.

9. Routine monitoring of tumor progression.
  a. From 1-week post-injection onward, perform visual and physical examinations 3 times per week to assess overall condition and tumor progression.
    ***Note:*** Tumor burden monitoring can be followed with bioluminescence imaging (BLI) if the injected cells contain a luciferase construct or using an MRI machine. However, routine BLI or MRI monitoring is optional, as humane endpoints can be determined through regular welfare assessments.
  b. Score each mouse based on general activity, appearance, behavior, body weight, body posture, neurological signs, and surgical site appearance to determine discomfort level.
  c. Record all measurements and observations for each mouse to monitor changes over time and detect early signs of tumor development.
    ***Note:*** Perform assessments at consistent time points to reduce variability between measurements.

10. Increased monitoring upon signs of deterioration.
  a. If early signs of tumor development or decline are observed (e.g., weight loss, decreased activity, altered gait), increase monitoring frequency to daily visual and physical examinations.
  b. Compare observations to previous records to evaluate progression and determine whether humane endpoint criteria are met. Early signs are typically observed 2–3 days before the humane endpoint is reached, with overall survival ranging between 30 to 50 days following tumor injection (Figure 4D).

***Note:*** In pre-adolescent mice, compare body weight to age-matched growth curves and/or litter mates to accurately assess deviations and identify weight loss.

11. Humane endpoints and euthanasia criteria.
  a. Euthanize mice immediately if any of the following criteria are observed: Immobility, weight loss >20%, lack of grooming, hunched posture, head tilt >45 degrees, circling behavior, seizures.
  b. If organs do not need to be collected, euthanize mice according to institutional guidelines by inhalation anesthesia overdose followed by decapitation (<6 days) or gradual CO_2_ inhalation (>6 days), otherwise continue with next steps.

### Deep anesthesia induction and transcardial perfusion for tissue collection (terminal procedure)

**Timing: 20–30 min per mouse**

These steps describe induction of deep anesthesia, transcardial perfusion for blood cell flushing, and collection of organs for ex vivo analyses.

12. Induction of deep anesthesia.
  a. Administer ketamine (75 mg/kg) and medetomidine (1 mg/kg) via intraperitoneal injection to induce deep anesthesia.
  b. Wait 5 min and confirm anesthesia by absence of reflex to a toe pinch before proceeding.
13. Opening the chest cavity to expose the heart.
  a. Pin or fixate the mouse on a dissection board with limbs spread to provide access to the thoracic and abdominal cavities.
  b. Apply a small amount of ethanol to the abdomen to keep hair in place and prevent contamination of the organs.
  c. Make a midline incision over the chest and abdomen and remove the skin to expose the thoracic cavity.
  d. Cut and retract the diaphragm and ribs as needed to fully access the heart and major vessels.

***Note:*** The mouse may gasp reflexively, but anesthesia should be sufficient to prevent sensation.

14. Clearing with PBS.
  a. Locate the heart and apex and stabilize it with forceps.
  b. Fill a syringe with 20 mL PBS and insert the needle into the left ventricle.
  c. Slowly inject PBS until the heart swells.
    **CRITICAL:** Inject solutions slowly to avoid vascular rupture and ensure uniform perfusion.
  d. Make a small incision in the liver to allow outflow.
  e. Continue perfusion until the liver appears pale, indicating effective clearing.
    **CRITICAL:** Proper PBS clearing before snap-freezing is essential for optimal tissue preservation for transcriptomic analyses and for reducing autofluorescence in downstream immunofluorescence analyses. Incomplete perfusion may result in residual blood within the tissue, leading to increased background signal in imaging datasets and the presence of blood-derived transcripts that may confound downstream transcriptomic analyses. In addition, thorough and consistent perfusion is recommended to ensure comparability between samples.

15. Remove the whole brain including pons and proximal spinal cord and transfer to cold PBS.

**CRITICAL:** Avoid damaging the brain or applying traction to the pons during dissection, as this may compromise tissue integrity and affect downstream analyses. Proceed with snap freezing immediately after brain harvest and minimize the time between euthanasia and freezing to preserve RNA integrity. In our experience, tissue can be maintained on ice and snap frozen within 30–60 min of euthanasia without adversely affecting downstream applications.

### Snap freezing, OCT embedding, and cryostat sectioning of mouse brains for quality control and downstream analyses

**Timing: 3 days**

These steps describe the snap freezing and OCT embedding procedure of intact adult mouse brains at study endpoint, as well as the cryostat sectioning for the RNA quality control and sequential sectioning for snRNA-seq, Visium and cIF. Refer to the 10x Tissue preparation guide for more details and consult minor modifications detailed below.

16. Snap freezing of intact brain in Tissue-Tek OCT using an isopentane bath cooled with dry ice.
  a. Remove intact brain from falcon tube containing ice cold PBS.
  b. Tap dry the brain using Kimtech wipes and deposit the brain into a cryomold.
    ***Note:*** Write down the orientation of the brain in the cryomold to facilitate sectioning later on.
  c. Cover the brain with Tissue-Tek OCT without introducing any air bubbles.
  d. Move the cryomold to the isopentane bath until completely frozen.
  e. Store the cryomold at −80°C over-night or for long term storage.
17. Cryostat sectioning for RNA quality control using RNA integrity number (RIN).
  a. Remove the tissue block embedded in OCT from the −80°C and leave it in the cryostat chamber at −20°C for at least 30 min before starting the sectioning.
  b. Pre-chill microcentrifuge tubes and brushes in the cryostat.
  c. Remove the OCT block from the cryomold and position it on the cryostat stage with the inferior ventral side towards you for horizontal sectioning.
  d. Cut 10–12 sections of 10 μm thickness and remove excess OCT around tissue if necessary.
    **CRITICAL:** Excessive OCT can negatively impact downstream RNA quality assessment and should be reduced as much as possible.
  e. Gently transfer the sections to a pre-cooled microcentrifuge tube using a brush.
    **CRITICAL:** Avoid touching the side of the tube to ensure the section will not melt.
  f. Immediately place the tube on dry ice. Store at −80°C or continue directly with RNA isolation. Avoid any thawing to preserve RNA integrity.
  g. Before storage of the leftover tissue block, cover the sectioned area with a small layer of Tissue-Tek OCT and store the sample block at −80°C until further steps.
  h. Extract RNA using the Qiagen RNeasy Mini Kit following the manufacturer’s guidelines for animal tissues.
  i. Measure the RIN on a bioanalyzer machine and proceed with the next steps if the RIN value is above 7.
    ***Note:*** Fresh frozen tissue should have a RIN value between 7 and 10.

18. Cryostat sectioning for snRNAseq.
  ***Note:*** Before proceeding with cryosections collection for snRNA-seq, ensure the current sections contain sufficient tumor cells by checking for GFP signal under a conventional fluorescent microscope.
  a. Repeat steps 17a-e.
  b. Immediately place the tube on dry ice.
  c. Store the sections at −80°C for up to 1 week.
19. Cryostat sectioning of sequential sections for 10× Visium.
  a. Pre-cool Visium spatial slide in the cryostat for at least 20 min in a closed slide holder.
    **CRITICAL:** Avoid any dust buildup on the Visium slides during handling and temperature equilibration.
  b. Cut a 10 μm section directly adjacent to the last section used for snRNAseq.
    **CRITICAL:** Do not exceed a section thickness of 10 μm. Thicker sections may contain multiple cell layers within a single Visium capture area, reducing spatial resolution and potentially introducing errors in downstream deconvolution analyses.
  c. Place the section onto a capture area of a pre-equilibrated Visium Spatial slide by gently touching the section with the active surface of the slide.
    ***Note:*** If the brain sections are too big to fit in the Visium capture area, use a clean razor blade to score the tissue block to get appropriately sized sections. Check the Visium Tissue preparation guide for further details on optimal tissue transfer and positioning.
  d. Gently press a finger on the back of the capture area for a few seconds to ensure proper adhesion and transfer the slide to the cryobar to ensure fast freezing.
  e. Move the slide back to the slide holder and seal the lid with parafilm.
  f. Store the slide at −80°C for up to 4 weeks.

20. Cryostat sectioning of sequential sections for cyclic immunofluorescence.
  a. Pre-cool Microscope slide in the cryostat for 10 min in a closed slide holder.
  b. Cut a 10 μm section directly adjacent to the last section used for 10× Visium.
    ***Note:*** While not required for downstream analysis, collecting the cyclic immunofluorescence section within 10–30 μm of the Visium section facilitates comparison between transcriptomic and protein-level data by preserving similar tissue architecture and cellular composition across the two modalities.
  c. Place the section in the center of the microscope slide and gently press a finger on the back of the slide for a few seconds to ensure proper adhesion.
  d. Transfer the slide to the cryobar to ensure fast freezing.
  e. Move the slide back to the slide holder and seal the lid with parafilm.
  f. Store the slide at −80°C for up to 4 weeks.

### Single nuclei RNA sequencing

**Timing: 3–4 h for nuclei isolation and sorting; 1 day for library preparation**

This step describes the isolation of nuclei from fresh-frozen mouse brain cryosections, fluorescence-activated nuclei sorting, and immediate processing using the 10× Genomics Chromium Single Cell 3′ platform. In this workflow, nuclei are isolated from frozen brain tissue under cold conditions optimized to preserve RNA integrity and minimize debris and doublets.

**Figure 5:**
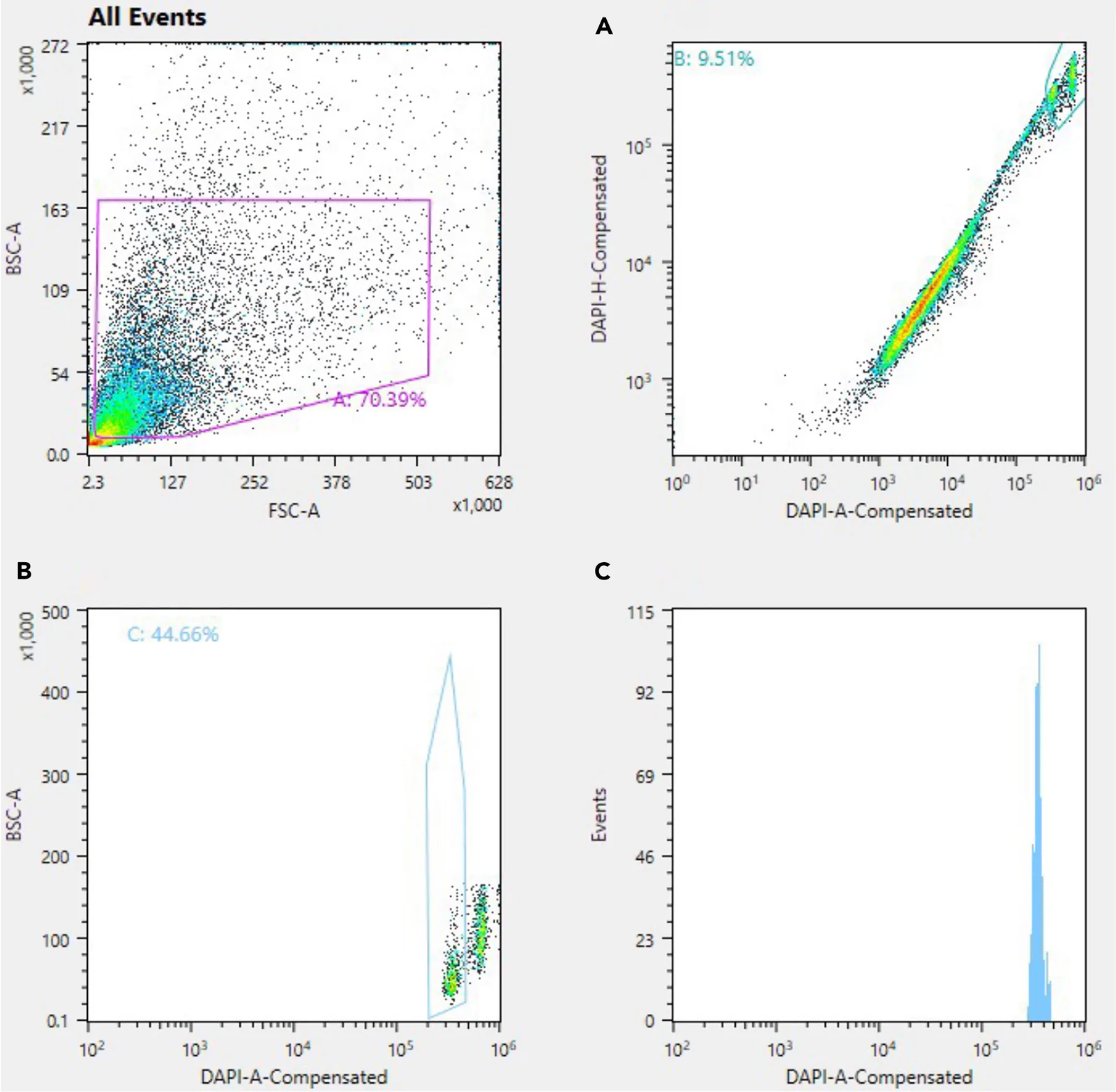
Representative FACS gating strategy for nuclei sorting Nuclei were first selected from all events based on FSC-A versus BSC-A to exclude debris (Gate A). Singlets were identified using DAPI area versus height (Gate B) and further refined by DAPI-A versus SSC-A to remove doublets and aggregates (Gate C), with a final DAPI histogram confirming a single homogeneous singlet population.
  c. Sort nuclei directly into chilled 10× RT buffer.
  d. Collect approximately 15,000 nuclei per tube for downstream Chromium Single Cell 3′ processing.
25. Chromium Single Cell 3′ library preparation.
  a. Immediately after sorting, add 8.3 μL RT Enzyme C to the sorted nuclei in RT buffer and mix gently.
  b. Load the sample onto the Chromium chip according to the Chromium Single Cell 3′ Reagent Kits User Guide from 10× Genomics.
  c. Proceed with GEM generation, reverse transcription, cDNA amplification, and library construction following the manufacturer’s instructions.
  d. Sequence libraries according to the Chromium Single Cell 3′ recommendations. For isolated nuclei, a minimum of 25,000 reads per nucleus is recommended; however, for brain tumor tissue, targeting 25,000–50,000 reads per nucleus is advisable to achieve sufficient gene detection depth. Perform downstream analysis using Cell Ranger with a pre-mRNA reference (including intronic reads), which is required for snRNA-seq data to capture unspliced nuclear transcripts.

***Note:*** The 10× Chromium platform generates doublets at an approximate rate of 1% per 2,000 captured nuclei (e.g., ∼6%–7% when targeting 15,000 nuclei). Computational doublet detection in brain snRNA-seq datasets typically identifies 3%–6% doublets. After quality-control filtering, a doublet rate below 10% is generally considered acceptable; samples exceeding this threshold should be flagged for potential re-isolation or exclusion.

21. Nuclei isolation from fresh-frozen tissue.
  a. Remove stored cryosections from −80°C storage and keep them on an ice-cooled metal block.
  b. Add 500 μL of chilled nuclei lysis buffer to the cooled microcentrifuge tube and gently pipette to dissociate the cryosections.
  c. Transfer the suspended cryosections in the chilled nuclei lysis buffer to a 2 mL dounce homogenizer.
  d. Homogenize the sample on ice using 5 strokes with the loose pestle, followed by 10 strokes with the tight pestle.
    **CRITICAL:** Do not twist the pestle to avoid nuclei shearing.
    ***Note:*** If a Dounce homogenizer is not available, nuclei can be isolated using optimized lysis buffer conditions combined with a short incubation time, eliminating the need for mechanical homogenization. This alternative approach has been described previously.^8^ Nuclei yield and quality may vary and should be validated for the tissue type and downstream application.
  e. Transfer the homogenate to a 2 mL DNA LoBind tube. Rinse the dounce with 1 mL chilled nuclei lysis buffer to recover remaining nuclei and combine this rinse with the original homogenate.
  f. Incubate the nuclei suspension on ice for 5 min, gently mixing once or twice during the incubation.
22. Nuclei washing and cleanup.
  a. Filter the homogenate through a 70 μm pipette tip strainer into a new 1.5 mL DNA LoBind tube.
  b. Centrifuge the nuclei at 500 × *g* for 5 min at 4°C. Carefully remove the supernatant, leaving approximately 50 μL above the pellet to avoid disturbing it.
  c. Gently resuspend the nuclei pellet in 0.5 mL wash buffer by pipetting up and down 10 times until no visible clumps remain.
  d. Inspect nuclei integrity and the presence of clumps microscopically.
  e. Add an additional 1.0 mL wash buffer to the nuclei suspension.
  f. Centrifuge the nuclei again at 500 × *g* for 5 min at 4°C. Carefully remove the supernatant without disturbing the pellet. If the pellet appears loose, leave approximately 50 μL residual volume.
  g. Resuspend the nuclei pellet in 300–500 μL wash buffer. Keep the suspension on ice until further processing.
  h. Inspect nuclei integrity and quality using a light microscope or cell counter.
23. Nuclei filtration and DAPI staining.
  a. Filter the nuclei suspension through a 40 μm pipette strainer immediately before sorting.
  b. Add 2.5 μL DAPI to 500 μL nuclei suspension.
  c. Keep the stained nuclei suspension on ice and proceed directly to fluorescence-activated nuclei sorting.
24. Fluorescence-activated nuclei sorting.
  a. Prepare chilled 10× RT buffer in advance, without adding RT Enzyme C.
  b. Identify single nuclei based on DNA content and exclude debris and aggregates during gating (Figure 5).

**CRITICAL:** Perform all steps on ice or at 4°C unless otherwise indicated. Avoid over-homogenization and minimize the time between nuclei preparation, sorting, and chip loading, as prolonged handling can compromise nuclei integrity and RNA quality.

### Spatial transcriptomics using 10× Visium

**Timing: 2 days**

This section describes the general steps necessary to run the 10× Visium spatial transcriptomic protocol with immunofluorescence staining using a DMG specific antibody combination on mouse brain sections.

26. Follow the Methanol Fixation, Immunofluorescence Staining & Imaging for Visium Spatial Protocol from 10× Genomics. Stain the sections with the DMG specific tumor marker (H3K27M, 1:400) and a general nuclei marker (DAPI, 1:2000).

***Note:*** Validate the antibody and concentration prior to using the Visium Slides to ensure they are compatible with methanol fixation and work with the 10× protocol.

27. Perform the tissue permeabilization, cDNA synthesis and library construction according to the protocol described in the Visium Spatial Gene Expression Reagent Kit User Guide.

***Note:*** For adult mouse brain sections of 10 μm we experimentally determined an optimal permeabilisation time of 16 min using the Visium Spatial Tissue Optimization protocol. Permeabilization time should be optimized based on tissue type and section thickness. Standard 10× Visium on fresh-frozen tissue should yield 1000–5000 genes detected per spot.

### Cyclic immunofluorescence and data acquisition

**Timing: 3–14 days depending on the number of imaging cycles**

These steps describe the preprocessing of brain cryosections on glass slides, the sequential primary and secondary antibody staining procedures, imaging on a fluorescent microscope or slide scanner and the antibody elution. Repeat this procedure as often as required to stain and image the complete marker panel required.

***Note:*** Before starting a cIF run, every antibody and marker combination should be validated on the tissue of interest using the same staining conditions. Ensure that weak markers are stained with strong secondary antibodies and that fragile epitopes like immune markers are imaged early in the imaging cycles. Do not combine two antibodies of the same species in the same imaging round.

28. Cryosection pre-processing for cIF.
  a. Remove unfixed cryosection from −80°C and thaw on a thermal cycler set at 37°C for 3 min. Proceed immediately to next steps.
  b. Draw a border around the tissue section using a hydrophobic barrier (PAP) Pen.
  c. Post-fix the cryosection by adding 4% PFA on top of the tissue area and incubate at room temperature for 10 min.
  d. Remove the PFA and submerge the glass slide in room temperature PBS for 5 min. Repeat the washing procedure with new PBS.
  e. Quench sample by adding 0.1 M Glycine on the tissue section for 20 min at room temperature.
  f. Wash the glass slide for 2 × 5 min in room temperature PBS.
  g. Add 0.2% Triton X-100 on top of the section for permeabilization and incubate for 10 min at room temperature.
    ***Note:*** Permeabilization conditions may require optimization depending on tissue type and section thickness. We recommend testing incubation times between 5–15 min. Excessive permeabilization may compromise the stainings.
  h. Wash the glass slide for 2 × 5 min in room temperature PBS.
  i. Store the glass slide in PBS overnight or proceed immediately with next steps.
29. Tissue blocking and primary antibody staining.
  a. Move glass slide to humidified staining chamber and add Blocking buffer to completely cover the tissue section area. Incubate at room temperature for at least 1 h and with gentle shaking.
  b. Dilute primary antibodies in Staining Buffer at a previously determined concentration.
    ***Note:*** Validate each primary antibody individually before multiplexing, including optimization of antibody concentration, confirmation of expected staining pattern, and assessment of background signal. Only antibodies with specific and reproducible staining should be included in the panel.
  c. Gently remove Blocking buffer with a pipette without touching the tissue and add the primary antibody mix. Incubate for 2 h at room temperature with gentle shaking.
  d. Remove glass slide from humidified chamber and wash for 3 × 5 min in room temperature PBS.
30. Secondary antibody staining and mounting.
  a. Dilute secondary antibodies and nuclei stain in Staining Buffer at a previously determined concentration.
  b. Centrifuge secondary antibody mix for 10 min at maximum speed (>10,000 rpm).
  c. Leave 50 μL at the bottom of the tube containing potential antibody aggregates and proceed with the remaining liquid.
  d. Move glass slide to humidified staining chamber and add Secondary antibody staining mix. Incubate for 2 h at room temperature and in the dark with gentle shaking.
  e. Remove glass slide from humidified chamber and wash for 3 × 5 min in room temperature PBS.
  f. Remove excess liquid around the tissue using a Kimtech wipe.
  g. Add 20 μL of mounting media to each section and slowly place a coverslip on top.

***Note:*** Do not use mounting media that solidify, avoid introducing air bubbles and ensure an even and thin layer of mounting media.

31. Imaging and coverslip demounting.
  a. Place glass slide in the fluorescent microscope.
    ***Note:*** Fluorescent microscope should be equipped with an automated stage, tile scan capabilities and able to image 4 fluorescent channels (405 nm, 488 nm, 555 nm, 647 nm). Ensure the air objective has sufficient resolution for intended downstream analysis and does not touch the glass slide at any step.
  b. Define a region of interest (ROI) slightly bigger than the actual tissue section and save in microscope settings for consecutive days.
  c. Define your focus map.
  d. Adapt exposure time and light source brightness according to the staining requirements and image the entire sample. Do not use Z-stacks.
  e. Review all images before removing sample from the microscope and save merged tile scans.
  f. Submerge glass slide in PBS at a 30° angle with the coverslip facing down.
  g. Let the coverslip slide off with gravity.
    ***Note:*** If coverslip does not fall off after several minutes to do not lift coverslip manually. Use tweezer to gently slide the coverslip from the tissue. Avoid damaging the tissue during de-mounting.
  h. Wash the glass slide for 2 × 5 min in room temperature PBS.
  i. Store the glass slide in PBS at 4°C over-night or until starting the next staining cycle. Do not exceed 48 h between cycles.
32. Antibody elution and tissue blocking.
  a. Prepare fresh elution buffer.
  b. Move glass slide to humidified chamber and add elution buffer to completely cover the tissue section area. Incubate at room temperature for 3 min with gentle shaking.
  c. Remove the elution buffer and wash the glass slide for 2 × 5 min in room temperature PBS.
  d. Repeat procedure from step 29 to 32 as many times as necessary to stain and image the planned marker panel.

***Note:*** Repeated staining and elution cycles may affect tissue integrity and epitope availability, leading to reduced antibody performance in later cycles (typically after >5 rounds). We recommend placing antibodies targeting sensitive or weakly expressed epitopes in early cycles and empirically determining antibody performance across cycles during panel optimization.

### Image co-registration

**Timing: ∼30 min setup; runtime depends on dataset size and computing power**

This section describes the steps required to install, set up, and execute the co-registration pipeline used to align consecutive imaging datasets from cyclic immunofluorescence experiments. The workflow is implemented in Python and is run through a Jupyter Notebook environment, enabling reproducible processing and generation of a final co-registered multichannel image output.

***Note:*** Computational requirements and runtime depend on image dimensions, bit depth, number of imaging cycles, and available hardware. Small demo datasets can be processed on a modern laptop with a multi-core CPU, SSD storage, and 32 GB RAM. However, full multi-day cyclic IF datasets require more memory and processing capacity, and were processed on a dedicated workstation with a 32-core CPU and 256 GB RAM.

33. Install required software.
  a. Install Miniconda or Miniforge.
  Option A: Install Miniforge (recommended) by downloading the appropriate installer for your operating system from the Miniforge GitHub releases page and follow the installation instructions.
  Option B: Install Miniconda by downloading the Miniconda installer for your operating system from the Miniconda downloads page and follow the installation instructions.
34. Download co-registration repository.
  Option A: Using Git (recommended).
  a. Open a terminal (or Anaconda Prompt on Windows) and run:
    >git clone https://github.com/Dream3DLab/CycFluoCoreg-STAR.git
    >cd CycFluoCoreg-STAR
    Option B: Download as ZIP (no Git required).
  b. Go to the GitHub repository page
  c. Click the green “Code” button
  d. Select “Download ZIP”
  e. Extract the ZIP file
  f. Open a terminal and navigate to the extracted folder:
    >cd path/to/extracted/CycFluoCoreg-STAR
35. Create the environment.

**Figure 6:**
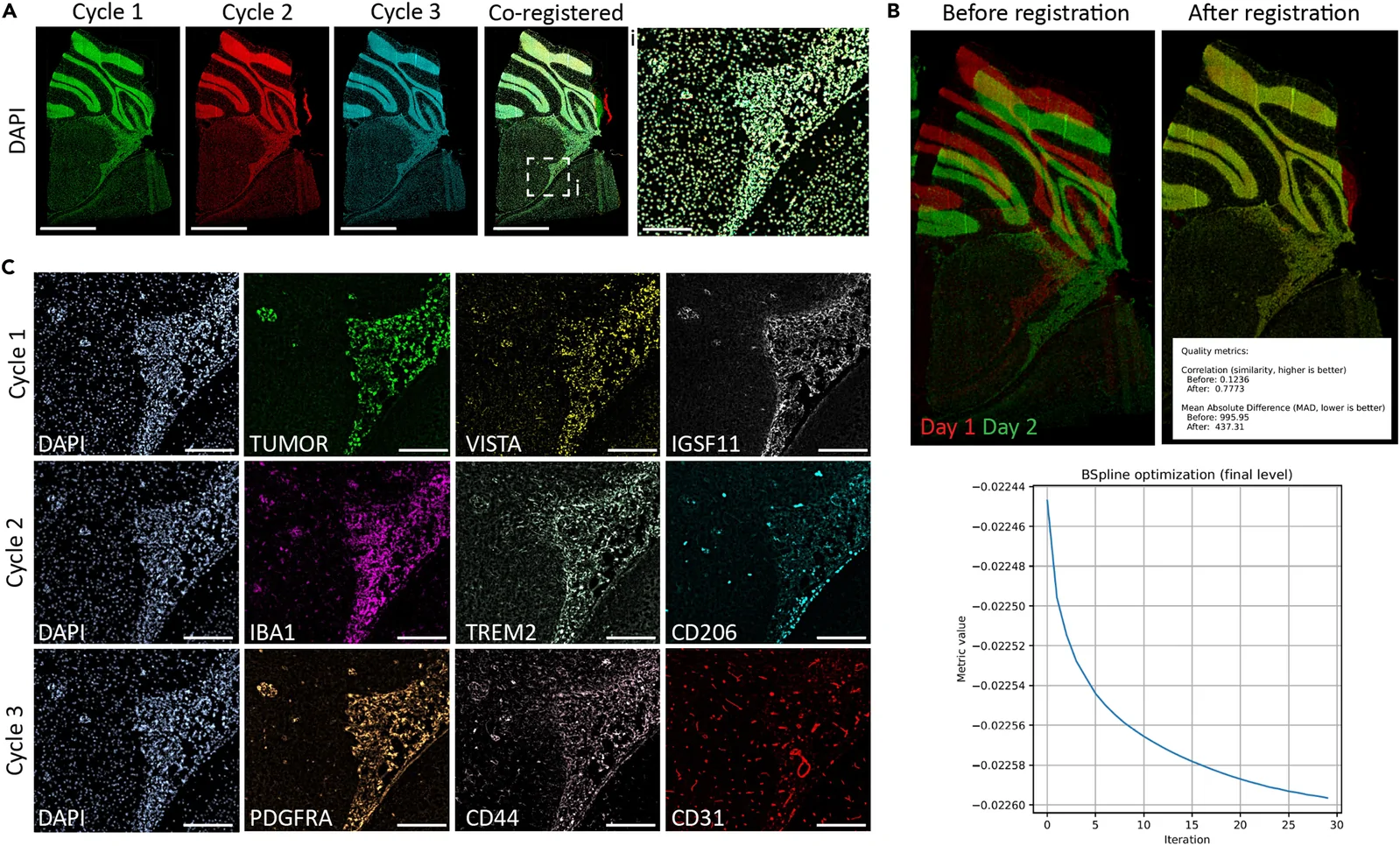
Co-registration of cyclic immunofluorescence images of a mouse DMG tumor (A) Visualization of DAPI staining in cycle 1, 2 and 3 of the cIF, next to the final co-registered file. Scale bar 2mm (full tissue) and 250 μm in zoom (i). (B) Example output of the quality control report after co-registration of Day 1 and 2. (C) 2D overview images of a mouse DMG tumor tissue slice stained for DAPI (blue, 1:2000), eGFP-Tumor (Green), VISTA (yellow, 1:200), IGSF11 (white, 1:100), IBA1 (magenta, 1:300), TREM2 (light green, 1:100), CD206 (cyan, 1:100), PDGFRA (orange, 1:400), CD44 (Light pink, 1:100) and CD31 (red, 1:200). Scale bar 250 μm.

>conda env create -f coregenvironment.yml

36. Activate the environment.

>conda activate coreg

37. Run the co-registration using a Jupyter Notebook in your browser.
  a. Launch Jupyter Notebook from the terminal.
    >jupyter lab
    ***Note:*** Jupyter notebooks may require authentication in your web browser before use.
  b. Navigate to the CycFluoCoreg-STAR.ipynb.
  c. Adjust the “Input paths” section at the top of the notebook according to your file locations.
  d. Optional: Adjust the “Analysis parameters” section at the top of the notebook.
  e. Run each step using Ctrl + Enter.
  f. Check quality control file when finished (Figure 6).
    **CRITICAL:** Ensure that all files are in .lif format, each file contains only one imaging day and that the tile scan within the file is merged. The Day 1 reference file must be stored separately and not included in the folder containing the remaining imaging days. File names should be identical across imaging days, differing only by the day number.

38. Visualize co-registered imaging file.
  a. Open the registered file in QuPath or equivalent software that supports multiplexed imaging files.
  b. Rename channels according to the corresponding markers (Figure 6).
  c. Visually verify correct alignment of imaging rounds by activating all DAPI channels.

## Expected outcomes

Following this protocol, we expect to obtain a comprehensive spatial characterization of the major cell types within the DMG tumor microenvironment, including key RNA and protein markers.

The generation of a DMG allograft mouse model at an early postnatal stage enables reproducible tumor formation in an immunocompetent setting and within a brain developmental context relevant to the disease. Intracardiac perfusion at the time of brain harvest, combined with rapid cryopreservation, ensures high-quality tissue suitable for downstream applications such as snRNA-seq and 10× Visium spatial transcriptomics, typically yielding RNA integrity numbers (RIN) above 7. From a single cryosectioned mouse brain, nuclei isolation (Step 21) is expected to yield sufficient material for the capture of approximately 7,000–10,000 nuclei per 10× Chromium run. High-quality snRNA-seq data should contain ≥200 detected genes and ≥500 UMIs per nucleus, with a post-filtering doublet rate below 10%. These data enable robust cell-type annotation and can be used to deconvolute Visium spatial transcriptomic spots from matched tissue sections. For DMG samples, a sequencing depth of 25,000–50,000 reads per nucleus is recommended to achieve adequate gene detection across the heterogeneous tumor microenvironment.

For 10× Visium, each capture area accommodates a tissue section up to 6.5 × 6.5 mm. Depending on tissue size and placement, approximately 3,000–5,000 spots are expected to overlay tissue. Given the 55 μm spot diameter, each spot typically captures transcripts from approximately 1–10 cells. Sequencing at ≥25,000 reads per spot is recommended to achieve sufficient transcript detection for downstream spatial analyses. These transcriptomic profiles, together with prior knowledge from the literature, can guide the design of antibody panels for cyclic immunofluorescence on sequential sections, enabling orthogonal validation.

Importantly, the staining, imaging, and co-registration workflow can be implemented using standard laboratory equipment and does not require specialized infrastructures.

## Quantification and statistical analysis

After generating snRNA-seq and spatial transcriptomics data, there is a wide range of software available in R and Python for downstream data processing, quantification, and statistical analysis.

For initial mapping and quantification of sequencing reads, we recommend using the software provided by 10× Genomics: Cell Ranger for snRNA-seq and Space Ranger for Visium spatial transcriptomics, as these tools handle alignment, barcode demultiplexing, and count matrix generation. Loupe Browser is available for interactive data exploration and visualization of both modalities.

For snRNA-seq preprocessing, clustering, and cell-type annotation, comprehensive frameworks such as Seurat and Scanpy are available, with SeuratWrappers enabling interoperability between the two. Quality control filtering criteria should be adapted to the tissue and experiment; in our dataset, nuclei with fewer than 200 detected genes, more than 5% mitochondrial reads, or identified as doublets by DoubletFinder were excluded. To distinguish malignant from non-malignant cells, copy-number variation inference can be performed using Numbat, which leverages allele frequency and expression-based signals to classify cells. This step is particularly relevant to tumor microenvironment studies, where distinguishing tumor from immune or stromal populations is critical.

For spatial transcriptomics analysis, spatial deconvolution tools such as Cell2location enable mapping of single-nuclei-defined cell states onto Visium spots and thereby linking transcriptomic profiles to spatial coordinates. Publication-ready visualizations can be generated using SCpubr and dittoSeq.

For cIF analysis, a free software like QuPath can be used for cell identification, cell type classification using machine learning and data extraction. Detailed multiplexed imaging analysis guidelines are available online. Alternatively, the commercial software MACS iQ View (Miltenyi Biotec) can be used for this task. All extracted cell features were exported and analyzed in R Studio.

The analytical steps will ultimately depend on the specific study questions, tissue type, and pathological states under investigation; the tools listed here represent the combination that we found effective for characterizing the tumor microenvironment in diffuse midline glioma, but alternative or complementary tools may be equally suitable depending on the experimental design.

## Limitations

This protocol has several limitations. The small size of the neonatal mouse brain introduces variability in stereotaxic accuracy, which may result in inconsistent pontine injection sites. It also requires low injection volumes (<2 μL) and increases the risk of cell reflux, potentially affecting reproducibility. Furthermore, the accuracy of the procedure depends on careful stereotaxic positioning and handling, which may introduce variability between operators, particularly during the initial learning phase. Spatial transcriptomics using the standard Visium platform (55 μm spot size) provides limited resolution, with each spot capturing transcripts from multiple cells. This necessitates computational deconvolution, typically supported by complementary snRNA-seq data. Higher-resolution alternatives such as 10× Visium HD, 10× Xenium, or Bruker CosMx may overcome this limitation but require dedicated machines and do not always offer whole transcriptome readout.

The protocol is optimized for fresh frozen tissue to enable compatibility with snRNA-seq, Visium, and cyclic immunofluorescence. Of note, cIF signal quality is generally improved in fixed tissue that has undergone transcardial perfusion with fixatives. Emerging approaches such as 10× Genomics Flex and Visium HD enable transcriptomic analysis of fixed-frozen and FFPE-archived samples and may provide suitable alternatives. Finally, manual cyclic immunofluorescence involves repeated coverslip removal, increasing the risk of tissue damage and variability compared to automated platforms such as Lunaphore or MACSima.

## Troubleshooting

### Problem 1

Needle clogging during Hamilton syringe loading or stereotaxic injection (related to step 4).

### Potential solution

• Low cell viability can increase debris and clogging. Use cell suspensions with >80% viability, keep cells on ice, and resuspend thoroughly before loading.
• High cell concentration can promote clogging. Dilute the suspension and adjust injection volume accordingly (do not exceed 2 μL). Clean the syringe thoroughly before reloading.

### Problem 2

Pups do not recover after surgery (related to step 6).

### Potential solution

• Hypothermia is a major risk. Minimize procedure time, use heating pads before and after surgery, and avoid excess liquids on pups.
• Monitor breathing and adjust anesthesia accordingly.
• Ensure accurate injection coordinates, as pontine damage can impair respiration.

### Problem 3

Low RNA quality (RIN) for downstream analysis (related to step 17).

### Potential solution

• Avoid sections with excessive necrosis or OCT.
• Minimize time from euthanasia to perfusion and snap-freezing.
• Maintain RNase-free conditions by cleaning tools, surfaces, and using sterile consumables.

### Problem 4

Cryosections crumble or fold (related to step 18–20).

### Potential solution

• Equilibrate tissue blocks at −20°C for 30–60 min before sectioning.
• Optimize cutting temperature (start at ∼−12°C for brain tissue and adjust as needed).
• Practice with training blocks and optimize mounting on standard slides before using Visium slides.

### Problem 5

Low nuclei recovery due to pellet loss, filtration clogging, or insufficient sorted volume (related to steps 22–24).

### Potential solution

• Nuclei pellets may not be visible at the bottom of the tube when using a fixed-angle rotor and can instead adhere to the tube wall. Always track tube orientation after centrifugation, aspirate supernatant from the opposite side, and gently rinse the expected pellet area to maximize recovery.
• During filtration, resistance may indicate clumping or debris. Do not force the suspension through the strainer; instead, gently resuspend and re-filter. If clumping persists, repeat the wash step prior to filtration.
• Final nuclei volume after sorting depends on instrument settings and nozzle size. Measure the collected volume and, if needed, adjust with chilled RT buffer (without enzyme C) to meet Chromium input requirements. Perform pilot runs to calibrate expected sort volumes for your setup.

### Problem 6

Tissue damage or detachment during cyclic immunofluorescence (related to step 28–32).

### Potential solution

• Increase drying time at 37°C (2–3 min) to improve adhesion.
• Use high-adhesion slides.
• Handle gently during staining and washing; avoid direct contact of the pipette tip with tissue.
• Discard folded or damaged sections before processing.

## Resource availability

### Lead contact

Further information and requests for resources and reagents should be directed to and will be fulfilled by the lead contact, Anne C. Rios (a.c.rios@prinsesmaximacentrum.nl).

### Technical contact

Technical questions on executing this protocol should be directed to and will be answered by the technical contact, Raphaël Collot (r.v.u.collot@prinsesmaximacentrum.nl).

### Materials availability

This study did not generate new unique reagents.

### Data and code availability

• Sequencing datasets generated as part of the original study^1^ are available on Zenodo: https://doi.org/10.5281/zenodo.21513336.
• Code for the image co-registration (CycFluoCoreg-STAR) can be found on GitHub, along with a tutorial dataset (https://github.com/Dream3DLab/CycFluoCoreg-STAR).

## Acknowledgments

All imaging was performed at the Princess Máxima Imaging Center and the mouse work at the Princess Máxima Animal Facility. This work was financially supported by the Princess Máxima Center for Pediatric Oncology and Oncode Institute, the Netherlands. R.C. and A.C.R. were supported by an ERC-starting grant 2018 project (no. 804412). The graphical abstract was created using Biorender.com.

## Author contributions

R.C. and A.C.R. designed and oversaw the study. R.C. and C.H. developed and performed the mouse experiments. C.R.-M. developed the snRNA-seq protocol, and C.R.-M. and R.C. performed the snRNA-seq and Visium ST experiments. C.R.-M. analyzed the transcriptomic data and developed the multi-omics integration pipeline. R.C. performed the cyclic immunofluorescence experiments and R.L.v.I. developed the co-registration pipeline. A.C.R. secured funding and R.C., C.H., and C.R.-M. wrote the protocol.

## Declaration of interests

The authors declare no competing interests.
